# Therapeutic Potential of Infrared and Related Light Therapies in Metabolic Diseases

**DOI:** 10.3390/ijms26115134

**Published:** 2025-05-27

**Authors:** Agnieszka Nowacka, Maciej Śniegocki, Wojciech Smuczyński, Ewa Ziółkowska

**Affiliations:** 1 Department of Neurosurgery, Collegium Medicum in Bydgoszcz, Nicolas Copernicus University in Toruń, ul. Curie Skłodowskiej 9, 85-094 Bydgoszcz, Poland; a.nowacka@cm.umk.pl (A.N.); sniegocki@cm.umk.pl (M.Ś.); 2 Department of Physiotherapy, Collegium Medicum in Bydgoszcz, Nicolas Copernicus University in Toruń, ul. Techników 3, 85-801 Bydgoszcz, Poland; w.smuczynski@cm.umk.pl; 3 Department of Pediatrics, School of Medicine, Washington University in St. Louis, St. Louis, MO 63110, USA

**Keywords:** far-infrared light therapy (FIR), metabolic diseases, oxidative stress, non-pharmacological therapies, inflammation, cardiovascular health

## Abstract

Infrared and related light therapies are gaining increasing interest due to their potential therapeutic properties in treating various health conditions, particularly metabolic diseases such as insulin resistance and type 2 diabetes. These diseases often coexist with dyslipidemia, obesity, non-alcoholic fatty liver disease, and cardiovascular complications. This review paper analyzes the impact, primarily of far-infrared light therapy (FIR), on improving endothelial function, reducing oxidative stress, and modulating inflammatory responses—key factors in metabolic diseases. Preliminary studies suggest that FIR may improve blood circulation, increase the secretion of VEGF, and enhance insulin sensitivity by alleviating inflammatory states and oxidative damage commonly associated with these diseases. In addition, FIR has been associated with potential benefits in blood pressure regulation and lipid metabolism, which could contribute to reduced cardiovascular risk. However, it is important to acknowledge that most current evidence is derived from preclinical models and small-scale clinical trials, limiting direct applicability to broader patient populations. Moreover, significant variability exists in exposure parameters and treatment protocols across studies. While FIR therapy holds potential as a complementary approach to the conventional management of metabolic diseases, careful monitoring is essential to mitigate potential adverse effects. Further well-designed, large-scale clinical trials are necessary to validate the therapeutic efficacy, optimize treatment parameters, and comprehensively assess the safety profile of FIR interventions in metabolic health.

## 1. Introduction

The modern world faces a growing challenge with the increasing prevalence of metabolic diseases, including obesity, insulin resistance, dyslipidemia, and cardiovascular diseases. These co-existing conditions often contribute to one another, impacting patients’ quality of life and posing serious health risks [1,2,3]. Metabolic diseases represent a major public health concern globally not only due to their high prevalence but also because of their complex pathophysiology involving genetic, environmental, and lifestyle factors. These diseases contribute significantly to morbidity and mortality rates worldwide, with type 2 diabetes and cardiovascular disorders ranking among the leading causes of death. In most cases, one of the first noticeable signs of metabolic disease is excess weight gain. According to the WHO, in 2022, there were 2.5 billion overweight adults globally, and this number continues to rise [4,5]. Pharmacological treatments for metabolic diseases frequently come with side effects and substantial economic costs, both in terms of therapy expenses and indirect costs such as lost productivity [6,7,8,9,10]. Consequently, there is an urgent need for effective, safe, and affordable preventive and therapeutic strategies to alleviate the burden of these conditions. Using natural approaches to prevent or manage these diseases is significant for both patients and national economies.

Metabolic diseases are influenced by various factors, including sedentary lifestyles, unbalanced diets, hormonal imbalances, and environmental factors, often leading to the development of low-grade inflammatory states [3,11]. Chronic inflammation is increasingly recognized as a central mechanism driving metabolic dysfunction, promoting insulin resistance, endothelial damage, and dysregulated lipid metabolism. These inflammatory states are associated with the presence of oxidative stress in cells and an imbalance between reactive oxygen species and the cell’s detoxification capabilities, ultimately resulting in cell damage [12,13,14]. Mitochondria play a crucial role in this process, as they are the site of reactive oxygen species production, which results from the functioning of the electron transport chain in the inner mitochondrial membrane [15,16,17]. Mitochondrial dysfunction further exacerbates oxidative stress, impairs energy metabolism, and contributes to the progression of metabolic diseases, making it a critical target for novel therapeutic interventions. This underscores the importance of treatments targeting the cellular level and the fundamental biological processes underlying these diseases. One natural way to stimulate cellular activity is through infrared light irradiation [18,19,20,21].

This review addresses a critical gap in the current literature by systematically analyzing the role of far-infrared and related light therapies in the context of metabolic diseases. While isolated studies have suggested the potential benefits of light-based therapies, a comprehensive, mechanistic understanding integrating photobiology, oxidative stress modulation, vascular biology, and metabolic regulation has been lacking. By highlighting the interdisciplinary convergence of photomedicine, molecular biology, and metabolic disease research, this review provides a translational perspective on how non-pharmacological light therapies could complement conventional interventions.

Specifically, this review focuses on summarizing the latest knowledge regarding the use of infrared and related light therapies, with particular emphasis on far-infrared radiation, in the prevention and management of insulin resistance, type 2 diabetes, lipid metabolism disorders, cardiovascular diseases, and inflammatory processes.

Additionally, this review discusses the limitations of current research, including the predominance of preclinical evidence and variability in study designs, and highlights the necessity of well-structured, large-scale clinical trials to validate therapeutic efficacy. The potential risks associated with infrared therapy, such as thermal injuries and ocular damage, are also considered, emphasizing the importance of cautious clinical application and patient monitoring in future translational efforts.

## 2. Mechanisms Underlying Far-Infrared Light Therapy

### 2.1. Spectral Characteristics of Infrared Radiation

**Figure 1:**
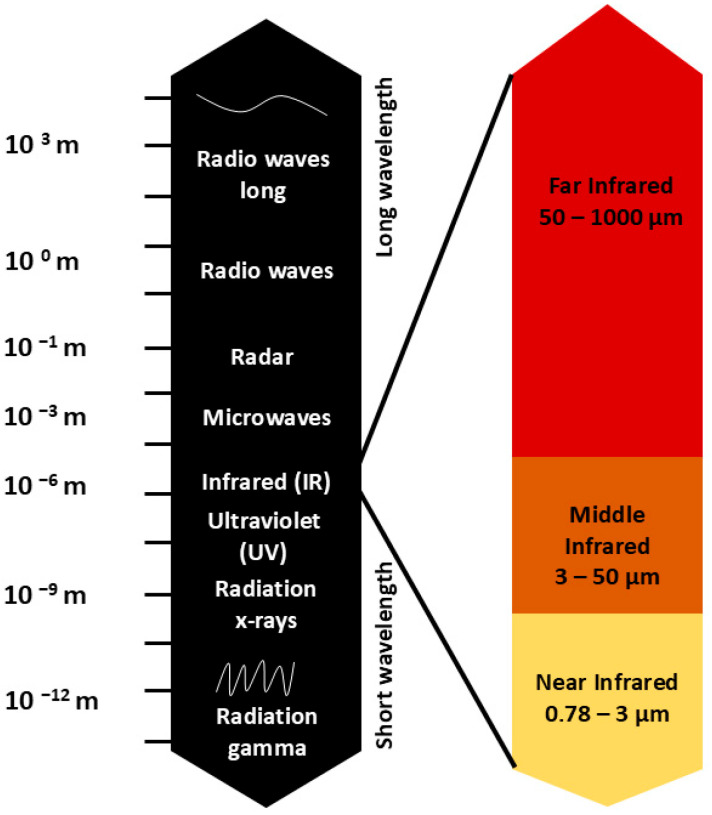
The electromagnetic spectrum and infrared radiation subdivision.

Infrared light is a type of electromagnetic radiation with wavelengths ranging from 0.78 to 1000 μm, further divided into three parts: near-infrared (0.78–3.0 μm), mid-infrared (3.0–50 μm), and far-infrared (50–1000 μm) (Figure 1) [22].

The figure illustrates the electromagnetic spectrum, spanning from high-energy gamma rays to low-energy radio waves. A detailed section highlights the subdivision of infrared (IR) radiation into near-infrared (0.78–3 μm), middle-infrared (3–50 μm), and far-infrared (50–1000 μm), showing their respective wavelength ranges.

While infrared light is not visually perceivable, it is experienced as heat, and its biological and therapeutic effects have been extensively studied. However, in many scientific publications, the middle- and far-infrared ranges are often combined and referred to collectively as “far infrared”, covering the range from 3 μm to 1000 μm. Moreover, in clinical studies investigating the effects of infrared light on human health, red light from the visible spectrum (0.62–0.75 μm) is often included as well, as it exhibits similar therapeutic properties. In this context, lamps used in therapy may emit both red and infrared light, covering a range from visible red light to far infrared. These devices are widely available on the market, combining both ranges of radiation into a single therapeutic technology [22,23,24,25].

It is important to note that other light-based therapies, including near-infrared (NIR) and visible light (particularly red light), have also demonstrated biological effects relevant to metabolic diseases. NIR penetrates tissues more deeply than far-infrared and has been shown to modulate mitochondrial function, reduce inflammation, and improve insulin sensitivity in various preclinical models. Similarly, photobiomodulation using red light (0.62–0.75 μm) influences cytochrome c oxidase activity and cellular metabolism, offering complementary mechanisms of action to far-infrared therapy. While this review focuses primarily on far-infrared radiation, acknowledging the therapeutic potential of these adjacent spectral regions enriches the translational landscape of light-based interventions in metabolic disease [22,23,24,25].

### 2.2. Biological Effects and Mechanisms of Action

Recent research indicates that infrared light can influence immunomodulation and promote tissue regeneration [20,26,27,28]. Irradiation with infrared light induces vasodilation, likely due to the regulation of vascular endothelial growth factor (VEGF) and the reduction in the antiangiogenic factor thrombospondin-2 (TSP-2) [19,20,29]. Increased microcirculation is crucial for delivering oxygen and nutrients to tissues and cells while also removing metabolic waste products. Previous analyses of the cellular mechanisms of infrared light have shown that the absorbed photon is converted into a signal that stimulates biological processes, including the activation of AMP-activated protein kinase (AMPK), cytochrome C oxidase, and intracellular water dynamics [30,31,32]. The changing water dynamics in cells and mitochondrial membranes under the influence of infrared light can impact intracellular metabolic processes, cytoskeleton organization, and cell proliferation. This effect is likely due to an increase in plasma membrane temperature and variability in electrical capacitance, leading to depolarization of the target cell [20,30,33,34,35]. In particular, far-infrared radiation (FIR) enhances mitochondrial function by stimulating electron transport chain complexes I, II, and IV, thereby improving cellular respiration and ATP synthesis, both of which are essential for metabolic processes and cellular regeneration. FIR-induced reactive oxygen species (ROS) production, at controlled levels, serves as a signaling mechanism that activates pathways involved in DNA repair and cell growth. However, excessive ROS can induce oxidative stress.

The molecular detection of infrared light in mammalian cells remains a topic of active research. Known photoreceptors such as cytochrome c oxidase (a component of mitochondrial complex IV) absorb photons primarily in the red and near-infrared spectra and initiate photobiomodulatory signaling cascades that enhance mitochondrial respiration and ATP production [21,24]. However, the mechanisms by which far-infrared wavelengths (>50 μm), which are primarily perceived as thermal radiation, exert specific biological effects remain less well defined. It is hypothesized that far-infrared radiation interacts with intracellular water molecules and membrane lipids, altering water cluster dynamics and membrane potential, thereby modulating cellular signaling indirectly rather than through classical photoreceptors [24]. Some studies suggest the involvement of opsin family proteins or other photosensitive molecules, but evidence is limited, and further research is necessary to clarify whether specific photoreceptors mediate FIR effects or if these are predominantly thermally induced biological responses.

### 2.3. Anti-Inflammatory and Regenerative Potential

**Figure 2:**
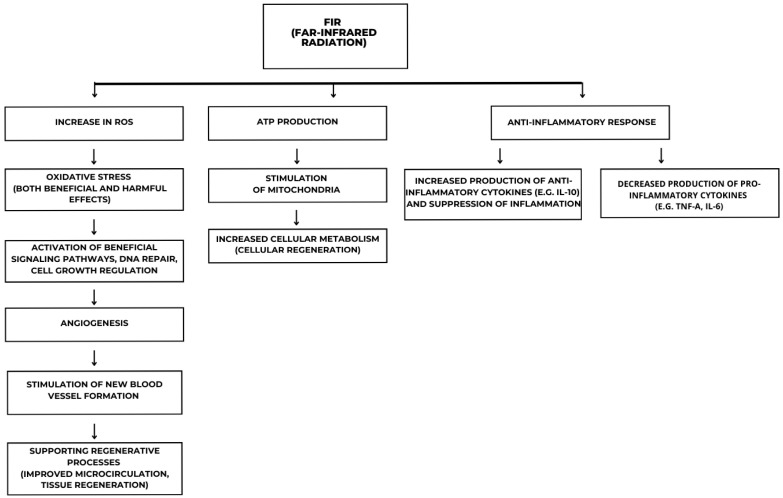
Molecular and cellular mechanisms induced by far-infrared radiation (FIR).

Additionally, FIR modulates inflammatory responses by activating the nuclear factor kappa-light-chain-enhancer of activated B cells (NF-κB) and mitogen-activated protein kinase (MAPK) pathways, leading to a decrease in pro-inflammatory cytokines like tumor necrosis factor-alpha (TNF-α) and interleukin-6 (IL-6), and an increase in anti-inflammatory cytokines like IL-10, effectively suppressing inflammation (Figure 2).

The figure illustrates the molecular and cellular mechanisms induced by FIR. FIR exposure leads to an increase in reactive oxygen species (ROS), triggering oxidative stress that can have both beneficial and detrimental effects. This stress activates key molecular signaling pathways involved in DNA repair, cell growth regulation, and mitochondrial function. The stimulation of mitochondria enhances ATP production and cellular metabolism, promoting regeneration and increased cellular activity. Additionally, FIR influences angiogenesis by inducing signaling cascades that support new blood vessel formation and tissue repair. Another crucial effect of FIR is its immunomodulatory role, characterized by an increased production of anti-inflammatory cytokines, such as interleukin-10 (IL-10), and a reduction in pro-inflammatory cytokines, including TNF-α and IL-6.

Analyses of both humans and animals have revealed a range of positive therapeutic effects from the use of infrared light (Table 1), including the treatment of hard-to-heal wounds, pain relief (including in rheumatoid arthritis), reduction in inflammatory conditions, and improvement in joint mobility [20,21,36,37]. It has also been shown to improve brain function in patients with depression and anxiety, with significant beneficial effects observed in patients with neurodegenerative diseases such as Parkinson’s and Alzheimer’s after infrared light therapy [38,39,40,41]. The high efficacy, low invasiveness, and safety of infrared light therapy, along with its ability to be combined with other methods, make it an attractive and significant approach for the prevention and treatment of metabolic diseases.

## 3. The Effect of Far-Infrared Light Therapy on Insulin Resistance and Type 2 Diabetes

### 3.1. Mechanisms Underlying Insulin Resistance in Type 2 Diabetes

**Figure 3:**
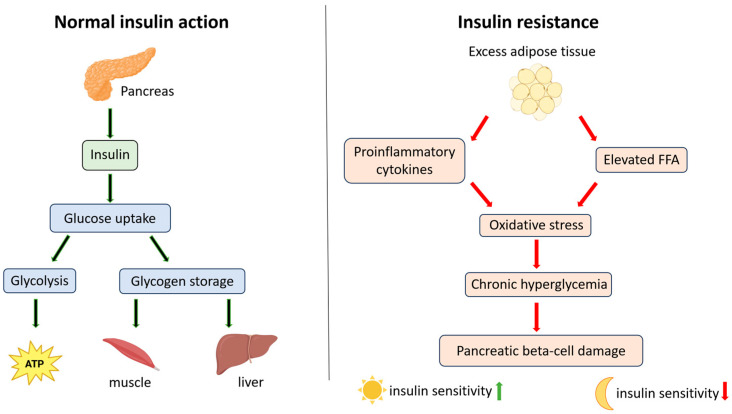
Mechanism of normal insulin action and insulin resistance. The diagram illustrates the differences between normal insulin signaling and the development of insulin resistance, highlighting the roles of pro-inflammatory cytokines, free fatty acids (FFAs), and oxidative stress.

The increasing prevalence of type 2 diabetes is a major cause of premature mortality worldwide [49,50,51,52]. Characterized by elevated blood glucose levels, type 2 diabetes is associated with impaired insulin secretion and tissue sensitivity to insulin [53,54]. Insulin, produced by pancreatic beta cells, binds to a tyrosine kinase receptor on various target cells [55,56,57,58]. This binding activates signaling pathways that promote the translocation of glucose transporters, particularly GLUT4, to the cell membrane, facilitating glucose uptake into cells. Other transporters, such as GLUT1, GLUT3, and GLUT12, may also play a role, with their insulin sensitivity varying by cell type and metabolic conditions. Inside the cell, glucose is utilized for ATP production via glycolysis or stored as glycogen in the liver and muscles [59,60,61,62,63,64,65]. In type 2 diabetes, insulin resistance develops, reducing tissue sensitivity to insulin. This resistance is associated with low-grade inflammation, with excess adipose tissue playing a key role. Adipocytes produce pro-inflammatory cytokines, including tumor necrosis factor-alpha (TNF-α), interleukin-6 (IL-6), and resistin, which impair insulin receptor activity and glucose transport [66,67,68,69,70]. Moreover, excess adipose tissue increases free fatty acids (FFAs) in the blood, intensifying oxidative stress and further disrupting insulin signaling. Long-term hyperglycemia can also lead to glucotoxicity, damaging pancreatic beta cells and exacerbating the metabolic dysfunction associated with type 2 diabetes [71,72,73,74]. Recent studies suggest that insulin sensitivity fluctuates throughout the day, influenced by factors such as light exposure, providing a basis for using light in the treatment or prevention of diabetes [75,76,77] (Figure 3).

### 3.2. Far-Infrared Light Therapy in Animal Models of Type 2 Diabetes

**Figure 4:**
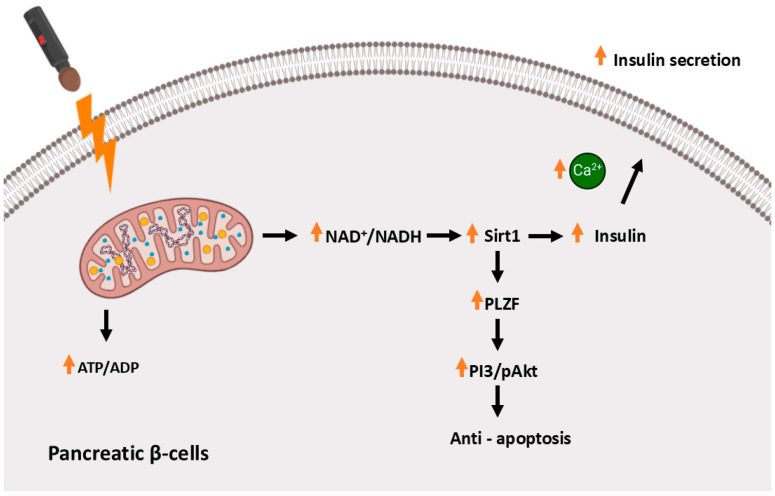
Mechanism of FIR in preserving pancreatic β-Cell mass and function. The figure illustrates the proposed mechanism by which far-infrared radiation protects pancreatic β-cells from dysfunction and apoptosis. FIR stimulates mitochondrial activity, leading to an increased ATP/ADP ratio and a higher NAD^+^/NADH level, which subsequently activates Sirt1. Sirt1 enhances insulin secretion and promotes cell survival through the activation of PLZF and the PI3K/Akt signaling pathway, reducing apoptosis. Additionally, FIR influences intracellular calcium (Ca^2+^) dynamics, further supporting insulin secretion and β-cell function. These mechanisms collectively contribute to the maintenance of β-cell mass and function under metabolic stress.

As reported by Hsu et al. [78], far-infrared light therapy significantly reduced fasting blood glucose levels in mice with NA-STZ-induced diabetes, a model that partially mimics type 2 diabetes by inducing beta-cell dysfunction rather than complete destruction. This suggests the potential application of this therapy in the treatment of metabolic disease. To analyze the effect of infrared radiation, the researchers irradiated the mice daily for 30 min over 1 and 2 weeks, using a wavelength range of 8–10 μm. As reported in the results, after two weeks of irradiation, both a decrease in glucose level and an increase in insulin levels were observed in the experimental group of mice and the positive control. Histological IHC analyses showed increased survival of pancreatic beta cells in the experimental groups, along with enhanced insulin production. Furthermore, the effect of FIR on the induction of the PLZF protein (promyelocytic leukemia zinc finger, ZBTB16) was demonstrated in the treated group compared to the control group. Studies suggest that PLZF may play a role in the protection of the vascular endothelium and also influence metabolism and oxidative stress [79,80,81]. It contributes to the regulation of metabolic pathways, including Sirtuin 1 (Sirt1), a NAD^+^-dependent deacetylase that plays a critical role in cellular metabolism, particularly in pancreatic beta cells. The activation of Sirt1 through FIR radiation increases the NAD^+^/NADH ratio, a key signal for enhancing mitochondrial function. The elevated NAD^+^/NADH ratio activates Sirt1, promoting the deacetylation of several substrates involved in mitochondrial biogenesis, oxidative stress response, and energy metabolism. These effects lead to enhanced mitochondrial ATP production, which is crucial for insulin secretion. Additionally, Sirt1 exerts anti-apoptotic effects in β-cells by inhibiting the expression of pro-apoptotic proteins and repressing uncoupling protein 2 (UCP2), a negative regulator of insulin secretion. By decreasing UCP2, Sirt1 enhances ATP generation, further supporting insulin release. Moreover, Sirt1 interacts with PLZF, enhancing its expression and promoting its anti-apoptotic effects. PLZF, in turn, plays a vital role in maintaining β-cell mass by protecting cells from oxidative stress and apoptosis, both of which are crucial mechanisms in the context of diabetes. The interaction between Sirt1 and PLZF creates a synergistic pathway that not only protects β-cells but also promotes their function and insulin production. In the next phase of the experiment, after 60 min of FIR irradiation, the researchers demonstrated an increase in mitochondrial function in RIN-m5f cells, along with an increase in the NAD^+^/NADH ratio in these cells. Therefore, the action of FIR light protected pancreatic beta cells from apoptosis while simultaneously stimulating insulin production and secretion (Figure 4).

### 3.3. Protective Effect of FIR Therapy on Endothelial Cells and AGE Accumulation

In hyperglycemic states, the glycation process is intensified, leading to the accumulation of advanced glycation end products (AGEs), which, in type 2 diabetes, can damage the vascular endothelium [72,73,82]. Furthermore, the accumulation of AGEs, through binding to the receptor for advanced glycation end-products (RAGE), can activate pro-inflammatory pathways and induce oxidative stress [83,84,85]. Chen et al. [80] investigated the effect of FIR light on AGE-induced endothelial damage using both in vitro and in vivo analyses. In vitro, human umbilical vein endothelial cells (HUVECs) were irradiated for 30 min at an intensity of 0.13 mW/cm^2^. In the in vivo experiments, the same irradiation parameters were applied to mice for 30 min a day over a period of 2 weeks. As reported, FIR irradiation inhibited AGE-induced apoptosis in human cells. This was accompanied by the activation of PLZF and an increase in the expression of phosphoinositide 3-kinases (PI3K), a key player in the autophagy signaling pathway (Figure 3) [86]. Immunofluorescence analyses revealed increased degradation of AGEs in HUVECs, facilitated by their transport to lysosomes. In the in vivo experiment using a diabetic mouse model, the researchers demonstrated a decrease in the concentration of AGEs in the blood serum following FIR light application, accompanied by a reduction in AGE deposition in the vascular endothelium. Furthermore, there was a decrease in the levels of inflammatory markers.

### 3.4. The Role of Red Light in Glucose Metabolism in Humans

Even short-term exposure to light with a wavelength of 0.67 μm, which corresponds to the red end of the visible light spectrum, has been shown to lower blood glucose levels. Powner and Jeffery [87] conducted a study investigating the effect of 0.67 μm wavelength light on glucose levels in 30 healthy volunteers. The participants were divided into two groups, experimental and placebo. An oral glucose tolerance test (OGTT) was performed at the beginning of the study, and the experimental group was irradiated for 15 min prior to glucose intake. Glucose levels were measured from capillary blood obtained through finger pricking. The results demonstrated that the PBM intervention significantly reduced glucose levels in the experimental group. Specifically, during the OGTT, there was a 7.9% decrease in total blood glucose concentration, with a *p*-value of 0.0012, indicating strong statistical significance. Additionally, when considering the absolute change in blood glucose concentration, the experimental group showed a 27.7% reduction in glucose level increase compared to their baseline measurements. This study also monitored exhaled carbon dioxide levels (etCO_2_), which exhibited significant differences between the experimental and placebo groups, suggesting a potential metabolic response to light exposure. This study suggests that the observed reduction in blood glucose levels is likely associated with increased mitochondrial activity in humans, a finding that has been consistently demonstrated both in vitro and in vivo in animal models [75,88,89,90]. Powner and Jeffery [87] also suggest that the action of 0.67 μm light increases mitochondrial potential and adenosine triphosphate production (ATP), which may increase glucose demand and its utilization by cells. In summary, this study indicates that light therapy could be a non-invasive intervention to help control blood glucose levels, particularly in individuals with impaired glucose homeostasis.

## 4. The Effect of Far-Infrared Light Therapy on Dyslipidemia and Non-Alcoholic Fatty Liver Disease

### 4.1. Role of the Gut–Liver Axis and MicroRNA Regulation in Dyslipidemia and Metabolic Dysfunction

Recent studies increasingly emphasize the pivotal role of the gut–liver axis in the pathogenesis of metabolic dysfunction-associated steatotic liver disease (MASLD) and related dyslipidemia. The intestinal microbiota exerts a significant influence on lipid metabolism, immune homeostasis, and hepatic function. Dysbiosis, defined as an imbalance in the composition and function of the gut microbial community, leads to disruption of the intestinal barrier integrity and increased permeability (“leaky gut”), allowing the translocation of bacterial endotoxins such as lipopolysaccharide (LPS) into the portal circulation. This event triggers Toll-like receptor 4 (TLR4)-mediated inflammatory cascades in Kupffer cells and hepatocytes, contributing to chronic low-grade inflammation, hepatic insulin resistance, and altered lipid metabolism, thereby promoting progression from steatosis to steatohepatitis and fibrosis [91,92].

Therapeutic modulation of the gut microbiome via probiotics, prebiotics, synbiotics, or specific dietary interventions (e.g., high-fiber and polyphenol-rich diets) has emerged as a promising adjunct strategy for MASLD management by restoring microbial diversity, enhancing the production of short-chain fatty acids (SCFAs) such as butyrate, known for their anti-inflammatory and hepatoprotective effects, and reducing endotoxemia [91,92].

MicroRNAs (miRNAs), short non-coding RNAs that post-transcriptionally regulate gene expression, play crucial roles in liver physiology and pathophysiology. Aberrant expression of specific miRNAs, including miR-122 and miR-34a, is associated with lipid accumulation, oxidative stress, inflammatory signaling, and fibrogenesis in MASLD. Increasing evidence supports their potential as diagnostic biomarkers and therapeutic targets in metabolic diseases [91,92].

In recent years, physical therapies such as far-infrared radiation (FIR) and low-level laser therapy (LLLT) have gained attention for their ability to modulate miRNA expression profiles. FIR therapy, by influencing cellular signaling pathways, can modify the expression of key miRNAs involved in lipid metabolism, inflammatory processes, and hepatic fibrosis. Preliminary studies suggest that epigenetic mechanisms induced by FIR may offer novel therapeutic avenues for dyslipidemia and MASLD, although further research is necessary to fully elucidate these effects [91,92].

### 4.2. Molecular Mechanisms Linking Insulin Resistance and Dyslipidemia

Dyslipidemia, characterized by abnormal blood lipid levels, increases the risk of cardiovascular diseases, including ischemic heart disease and atherosclerotic cardiovascular disease. It often progresses asymptomatically, making early detection through regular lipid profile testing essential [93,94]. The basic lipid profile includes the analysis of triglyceride concentration, cholesterol levels, low-density lipoproteins (LDLs), and high-density lipoproteins (HDLs). Factors contributing to the development of dyslipidemia include a sedentary lifestyle, an unhealthy diet leading to obesity, and genetic factors [95,96]. With over 500 million obese individuals worldwide, the growing prevalence of obesity presents significant health and economic challenges [97,98]. Current treatment strategies focus on lifestyle modifications, including diet and physical activity, and pharmacological interventions, such as the use of statins. Unfortunately, the pharmacological approach at every stage of the disease carries a risk of side effects. To avoid this, it is worth exploring natural methods, in addition to lifestyle changes, to support the body in its fight against the emerging metabolic disorder known as dyslipidemia.

Dyslipidemia arises from excessive very low-density lipoproteins (VLDLs) production, reduced LDL receptor expression, and impaired HDL function, often linked to insulin resistance. This leads to excessive lipolysis in adipocytes due to decreased insulin inhibition of hormone-sensitive lipase (HSL), which increases free fatty acid (FFA) release [99,100,101,102]. In the liver, excess FFAs stimulate the activity of sterol regulatory element-binding protein 1c (SREBP-1c), which induces triglyceride (TG) synthesis and expression of apolipoprotein B-100 (ApoB-100), a key component of VLDL [103,104]. Chronic inflammation driven by TNF-α and IL-6 further exacerbates insulin resistance and VLDL production, while macrophages in blood vessels convert oxidized LDLs into foam cells, promoting the development of atherosclerosis [105,106]. Additionally, macrophages exhibit impaired function of the cholesterol transport protein ABCA1, limiting the transfer of cholesterol to HDLs and weakening the reverse cholesterol transport mechanism to the liver. Consequently, decreased LDL uptake by the liver, impaired HDL function, chronic inflammation, and impaired VLDL catabolism can lead to dyslipidemia. This, in turn, can progress to metabolic dysfunction-associated steatotic liver disease (MASLD/NASH), ultimately increasing the risk of cardiovascular diseases [107,108,109].

### 4.3. Pathogenesis of Non-Alcoholic Fatty Liver Disease

Non-alcoholic fatty liver disease is a spectrum that ranges from simple fatty liver to more advanced stages, which can progress to liver fibrosis, cirrhosis, and, with a high probability, the development of hepatocellular carcinoma (HCC) [110,111,112].

It develops as a result of the aforementioned interaction of insulin resistance, excessive influx of free fatty acids, disrupted lipogenesis, and the onset of chronic inflammatory states [107,113]. In hepatocytes, hepatosteatosis occurs due to the elevated level of FFA circulating in the bloodstream, caused by insulin resistance. Additionally, in the mitochondria, β-oxidation of fatty acids is impaired, further deepening their accumulation. The lipid excess leads to lipid peroxidation and the production of reactive oxygen species, which activate oxidative stress. Consequently, this increases pro-inflammatory cytokines, recruiting inflammatory cells and activating Kupffer cells, which exacerbate hepatocyte damage [13,114,115]. The long-term intensification of pro-inflammatory factors and oxidative stress activates hepatic stellate cells (HSCs), which, under the influence of TGF-β, promote liver fibrosis. This process leads to the progression of MASLD towards non-alcoholic steatohepatitis (NASH) and eventually cirrhosis and hepatocellular carcinoma [107,108,113,116].

### 4.4. Far-Infrared Light Therapy in Animal Models of Dyslipidemia

Previous studies have shown that infrared radiation significantly modulates lipid metabolism in various cells of the organism. This modulation occurs through the enhancement of enzymatic processes, improved insulin sensitivity, and the formation of lipid droplets, which collectively contribute to better fat metabolism [76,117].

To analyze the effect of infrared light on metabolic dysfunction-associated fatty liver disease (MAFLD), Xu et al. [118] used a mouse model that was systematically irradiated with FIR light in the abdominal region every other day for 4 weeks. This study showed that FIR treatment significantly reduced hepatic lipid accumulation in the experimental group, as confirmed by histopathological analysis and biochemical tests that showed lower levels of triglycerides and total cholesterol in the livers of FIR-treated mice compared to control groups. Further, serum analysis revealed a decrease in TG, TC, and low-density lipoprotein cholesterol, along with an increase in high-density lipoprotein cholesterol in the FIR-treated groups. This suggests an overall improvement in lipid profiles due to FIR therapy. In the further part of the experiment, RT-qPCR analyses showed that FIR treatment led to the downregulation of genes associated with lipogenesis (CD36, FASN, PDK4) and the upregulation of genes related to fatty acid oxidation. These findings were further supported by Western blot analyses, highlighting the molecular mechanisms by which FIR exerts its effects. This study hypothesized that FIR treatment reduces lipid deposition through the activation of the AMPK signaling pathway. After FIR treatment, increased phosphorylation of AMPK was observed in the liver tissue, promoting metabolic activity and contributing to the alleviation of MAFLD. In addition to the in vivo results, FIR treatment also demonstrated a protective effect against MAFLD in vitro in hepatocytes, further supporting its potential as a therapeutic approach for this condition. These results collectively illustrate the promise of FIR as a new therapeutic strategy for the treatment of metabolic dysfunction-associated fatty liver disease.

### 4.5. Clinical Evidence: FIR Therapy in Individuals with Dyslipidemia

In another study, the researchers analyzed the effect of FIR light on various parameters of individuals with dyslipidemia. This study invited a group of three men and one woman with dyslipidemia, and a control group of the same size, in the age range of around 50 years. The volunteers were irradiated once a week for 15 min, with FIR light, with a wavelength of 9–12 μm and an output power of 30 mW, applied at four points on the body. The experiment lasted for 3 weeks. Lipid profile analysis in patients with dyslipidemia showed the following results: LDL-C before therapy was, on average, 151.3 ± 4.9 mg/dL, which decreased to 131.5 ± 9.1 mg/dL after therapy; triglyceride levels were 309.3 ± 154.4 mg/dL before therapy and 154.8 ± 30.5 mg/dL after therapy; and HDL-C did not undergo significant change and remained within the normal range. In the control group, no statistically significant changes were observed. This study clearly suggests a positive effect of FIR therapy on lipid metabolism in patients with dyslipidemia, indicating its potential as a therapeutic intervention [119].

The aim of the next study was to evaluate the combined effect of low-level laser therapy and a Mediterranean diet on older adults with non-alcoholic fatty liver disease. In a randomized, controlled clinical trial, 80 participants aged 65–75 years were divided into two groups of 40 each: one group received both laser therapy and dietary intervention, while the other group followed only the Mediterranean diet. After 12 weeks, key health indicators were assessed, including liver enzyme levels, lipid profile, and anthropometric parameters. The results showed that the combination of LLLT and MD led to a significant improvement in liver function and metabolic parameters compared to the diet alone. The average BMI reduction in the LLLT + MD group was 2.1 ± 0.5 kg/m^2^, while in the diet-only group, the BMI reduction was 1.4 ± 0.4 kg/m^2^. Triglyceride levels in the LLLT + MD group decreased from 190 ± 15 mg/dL to 140 ± 12 mg/dL, while in the MD group, they decreased from 185 ± 14 mg/dL to 155 ± 13 mg/dL. HDL concentration increased from 42 ± 5 mg/dL to 50 ± 4 mg/dL in the LLLT + MD group and from 43 ± 6 mg/dL to 47 ± 5 mg/dL in the MD group. LDL concentration decreased in the LLLT + MD group from 130 ± 10 mg/dL to 110 ± 8 mg/dL and in the MD group from 128 ± 9 mg/dL to 118 ± 8 mg/dL. Laser therapy at a wavelength of 0.66 μm was administered twice a week for 30 min, which may have contributed to the reduction in inflammation and the improvement in liver function. The carefully selected study group and rigorous methodology enhance the reliability of the results, suggesting that the combination of LLLT and MD represents an effective therapeutic strategy in managing NAFLD and reducing body mass [120].

In summary, the findings discussed in this section demonstrate the therapeutic relevance of far-infrared light therapy. Table 2 provides a concise overview of the most important data related to dyslipidemia.

## 5. Far-Infrared Light Therapy and Cardiovascular Diseases

### 5.1. Metabolic Dysregulation as a Risk Factor for Cardiovascular Diseases

**Figure 5:**
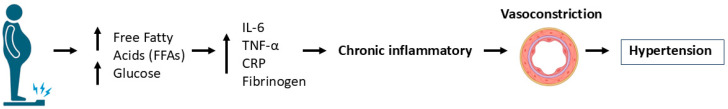
Pathophysiological mechanism linking obesity, inflammation, and hypertension. The diagram illustrates the pathway through which obesity contributes to the development of hypertension. Increased levels of free fatty acids (FFAs) and glucose lead to the upregulation of pro-inflammatory cytokines, including interleukin-6 (IL-6), tumor necrosis factor-alpha (TNF-α), C-reactive protein (CRP), and fibrinogen. The resulting chronic inflammatory state induces vascular dysfunction and vasoconstriction, ultimately leading to hypertension.

The metabolic diseases discussed in the previous chapters, such as insulin resistance, type II diabetes, and dyslipidemia, often accompanied by obesity, can lead to the development of cardiovascular diseases (Figure 5) [121].

Disturbances in glucose and lipid metabolism, the presence of oxidative stress, and existing inflammatory states contribute to the formation of blood vessel damage, atherosclerosis, and hypertension [118,122,123,124]. The key role is played by the dysfunction of the vascular endothelium, which regulates vascular tone, vessel permeability, and hemostasis [125,126]. Under optimal physiological conditions, the endothelium produces nitric oxide, which acts as an anti-inflammatory and vasodilatory agent. However, under the influence of the mentioned risk factors, there is often a decrease in NO synthesis, increased production of pro-inflammatory cytokines such as interleukin-6 and TNF-α, and the activation of adhesion molecules that facilitate the adhesion of monocytes to the vessel wall [125,127,128,129,130]. The described endothelial dysfunction, along with the onset of chronic inflammation, oxidative stress, lipid accumulation, and hypertension, consequently leads to the development of atherosclerosis [125,131]. Atherosclerosis results in the narrowing of the vessels and restriction of blood vessels and blood flow, which often leads to serious complications such as myocardial infarction, ischemic stroke, heart failure, aneurysms, and vessel ruptures [132,133,134,135,136,137,138]. Understanding these mechanisms facilitates the implementation of effective preventive and therapeutic measures, such as reducing risk factors (e.g., dietary changes, increasing physical activity) and often the use of lipid-lowering and antihypertensive drugs. Moreover, to promote better and faster recovery, it is also essential to explore alternative natural therapeutic agents that could support treatment and/or act preventively.

### 5.2. Effect of FIR Light Therapy on Blood Flow and VEGF Levels

The research group described in the previous chapter [119], which conducted an experiment on a group of four individuals with dyslipidemia, also examined the effect of FIR light on blood flow and the level of vascular endothelial growth factor (VEGF). VEGF is a protein that plays a crucial role in angiogenesis, acting on the endothelial cells of blood vessels. An increase in its level can occur in metabolic diseases and cancers, while a decrease often leads to tissue hypoxia [139,140,141]. Blood flow in the facial artery was analyzed using a laser Doppler flowmeter. Blood circulation in the retina—retinal arteries and veins—was assessed using laser speckle flowgraphy. Furthermore, blood analyses were performed to measure VEGF using an electrochemiluminescence method. The analyses showed an increase in blood flow in the retinal vessels of patients with dyslipidemia after FIR light irradiation. The average flow was 16.3 ± 4.7 mL before therapy, 20.3 ± 6.2 mL after the first FIR session, and 30.9 ± 14.6 mL after the second session. In the group of healthy volunteers, no significant changes in blood flow were observed, with an average value of 20 ± 4.5 mL. VEGF analyses showed a significant increase after therapy in patients, with the initial level not exceeding the upper limit of the normal range of 38.3 pg/mL, while in patients with an initial VEGF level above the norm, a decrease was observed. These results suggest a capacity for the use of FIR to improve blood flow in blood vessels and normalize VEGF levels.

### 5.3. Clinical Evidence: FIR Therapy in Patients with Cardiovascular Diseases

Furthermore, the results of Beever et al. [142] suggest that the use of far-infrared in the form of a sauna has a beneficial effect on lowering blood pressure. This study involved 14 women and men already suffering from type II diabetes. Participants underwent 20 min far-infrared sauna sessions three times a week for three months. Baseline measurements were taken within a week before the start of the sauna sessions, and post-intervention measurements were collected between 1 and 3 days after the last session. Analyses showed a significant decrease in systolic blood pressure of 6.4 mmHg, from 124 ± 12 to 118 ± 15 mmHg. A trend towards a reduction in waist circumference, with a decrease of 2.3 cm, was also observed. Although this study utilized a small sample size and considered findings with *p* < 0.10 as potentially significant, the results indicate a promising effect of far-infrared sauna use on cardiovascular health.

## 6. Potential Risks and Precautions with Far-Infrared Light Therapy

Infrared light therapy is gaining increasing attention as a supportive treatment modality in various clinical settings, including chronic pain, vascular dysfunctions, and inflammatory conditions. Nevertheless, like any therapeutic intervention, it is not without risks. The following subsections outline key adverse effects associated with FIR.

### 6.1. Cutaneous Effects and Photocarcinogenic Risk

Repeated or excessive exposure to infrared radiation can adversely affect the skin’s structural and molecular integrity. Studies have shown that IR may contribute to photoaging, primarily through increased expression of matrix metalloproteinases (e.g., MMP-1), leading to collagen degradation and oxidative stress [143,144]. Furthermore, preclinical data raise concerns about photocarcinogenesis, suggesting that chronic IR exposure may potentiate mutagenic processes at the cellular level [145]. Although direct evidence in humans is limited, this mechanism remains plausible and underscores the importance of limiting cumulative exposure in clinical settings.

### 6.2. Ocular Safety Concerns

The eyes are particularly vulnerable to infrared radiation due to the high transmissibility of IR through the cornea and lens. Clinical reports and safety assessments have documented potential risks including retinal phototoxicity, lenticular opacities, and visual disturbances, especially in cases where protective eyewear was not used [146,147,148]. For this reason, shielding of ocular structures is considered mandatory during FIR-based interventions, particularly in treatments involving the upper body or head and neck region.

### 6.3. Paradoxical Pain Sensitization

Although far-infrared (FIR) therapy is widely applied for pain relief, some reports suggest that it may paradoxically exacerbate pain sensitivity in certain individuals. The underlying mechanisms are not fully elucidated but may involve the activation of heat-sensitive nociceptors, such as TRPV1 channels, or the modulation of inflammatory mediators within affected tissues. While direct clinical evidence remains limited, some preclinical findings and theoretical considerations raise concerns regarding increased pain perception in patients with chronic pain syndromes, including fibromyalgia and complex regional pain syndrome. These considerations underscore the need for cautious, individualized application of FIR therapy, particularly in populations with dysregulated nociceptive processing [149].

### 6.4. Effects on Implanted Medical Devices

Due to its ability to penetrate deep tissues, FIR radiation may pose theoretical risks to individuals with implanted electronic medical devices, such as pacemakers or neurostimulators. Although FIR does not emit ionizing radiation, its thermal and electromagnetic properties have raised concerns regarding potential interference with device functionality. While systematic clinical evaluations are lacking, expert opinion and isolated case reports suggest that FIR therapy should be contraindicated in patients with such implants unless prior clearance is obtained from the device manufacturer or treating physician.

### 6.5. Cardiovascular Considerations

FIR therapy has been associated with vasodilatory effects and improved peripheral circulation, outcomes that may be beneficial in certain contexts. However, these hemodynamic changes could pose risks for individuals with unstable cardiovascular conditions, such as labile blood pressure or arrhythmias. Although some small-scale studies and anecdotal evidence have described improvements in vascular function with repeated FIR exposure, occasional episodes of hypotension and dizziness have also been observed. Given the lack of large-scale controlled trials, FIR therapy should be applied cautiously in patients with cardiovascular comorbidities, and vital parameters should be closely monitored [142,150].

### 6.6. Systemic Thermal Stress

Prolonged or whole-body exposure to FIR can induce systemic thermal effects, including elevated core body temperature, profuse sweating, and mild dehydration. These effects are particularly relevant in oncology patients, who may be more susceptible to electrolyte imbalance or fluid loss due to concurrent therapies. While FIR sessions improved subjective symptoms in cancer patients and those with chronic fatigue syndrome, a minority experienced orthostatic intolerance and thermal discomfort [149,150]. Proper hydration, session duration control, and pre-therapy screening are therefore essential components of safe practice.

### 6.7. Recommendations for Clinical Practice

Given the above considerations, FIR therapy should be administered with strict attention to safety protocols. This includes limiting session duration, monitoring patient response, and using protective equipment—particularly for the eyes. Individuals with pre-existing conditions, photosensitivity, or implanted devices should undergo a thorough medical evaluation prior to treatment initiation. Table 3 presents a summary of exposure parameters and safety recommendations derived from available clinical and experimental data.

The recommended safe exposure parameters presented in Table 1 are approximate values based on available preclinical and early clinical evidence. Variations may occur depending on the specific device, treatment protocol, and individual patient characteristics. Careful monitoring and adjustment are advised to ensure safe application.

## 7. Limitations and Future Directions

This review highlights a significant gap in the existing literature by systematically assessing the impact of far-infrared and similar light therapies on metabolic disorders. Although initial findings are encouraging, it is crucial to recognize several limitations. Most of the existing evidence is derived from preclinical models and small clinical trials, with a scarcity of large, randomized controlled studies validating therapeutic effectiveness and safety across broader patient groups. Additionally, considerable variability in study methodologies, including variations in wavelengths, exposure conditions, and treatment protocols, hinders comparisons and restricts the formation of standardized guidelines. The potential risks linked to extended exposure, such as thermal injuries and eye damage, necessitate further exploration, especially in susceptible patient populations. Future investigations should focus on well-structured, multicenter clinical trials to establish optimal exposure conditions, assess long-term effects, and validate the practical applicability of infrared and similar light therapies in treating metabolic disorders. Ongoing interdisciplinary collaboration among photomedicine, molecular biology, and clinical sciences will be vital to fully harness the therapeutic potential of these non-pharmacological approaches.

## 8. Conclusions

Infrared light therapy, particularly far-infrared radiation (FIR), shows potential in managing metabolic disorders by improving microcirculation, reducing inflammation and oxidative stress, enhancing vascular function, and regulating pro-inflammatory cytokines. These effects may lead to improved insulin sensitivity and lower risks of vascular issues like hypertension and atherosclerosis. FIR therapy may also promote angiogenesis, tissue regeneration, and better glucose uptake, while positively affecting lipid profiles by reducing LDL cholesterol and triglycerides. However, most evidence comes from preclinical studies and small clinical trials with varied designs, warranting caution in integrating FIR into standard practice. It should complement, not replace, established methods like a healthy lifestyle and exercise. Future research should focus on large-scale randomized controlled trials to verify FIR’s effectiveness and safety, along with standardizing treatment parameters for optimal results and understanding its mechanisms and long-term safety.

## Figures and Tables

**Table 1:** The use of infrared light in the therapy of various disorders.

Application	Target	Wavelength	Results	Ref.
Adipose Regeneration	Human adipose-derived stem cells	0.81 μm 0.98 μm	Promote cell proliferation and differentiation	[42]
Neural Stimulation	HEK-293T cells	1.889 μm	Altered the membrane electrical capacitance during optical stimulation transiently	[33]
In vivo models				
Wound Healing	Dermal abrasions in mice	0.81 μm	Increased collagen deposition and improved healing effects	[43]
Brain Neural Regeneration	Strokes in embolized rabbits	0.808 μm	Elevated ATP levels in the cortex	[44]
Neural Stimulation	Rat sciatic nerve	1.875 μm	Hybrid electro-optical stimulation induced sustained muscle contractions while lowering laser power requirements	[45]
Neural Stimulation	Adult rabbit heart	1.851 μm	Triggered optical pacing in the adult rabbit heart	[46]
Wound Healing	Soft tissues in rats	0.904 μm	Enhances wound healing and affects membrane properties, as measured by ^1^H-NMR τc\tau_cτc	[47]
Clinical model				
Brain Neural Regeneration	Mild traumatic brain injury	0.87 μm	Enhanced cognitive function, better sleep, and reduced symptoms of post-traumatic stress disorder	[48]

The table presents examples of the use of infrared light in tissue regeneration and neuronal stimulation. It summarizes information regarding target tissues, wavelengths, and the obtained effects. The studies are divided into preclinical (animal and cellular) and clinical models to better illustrate therapeutic applications.

**Table 2:** Summary of the effects and mechanisms of far-infrared (FIR) light therapy on dyslipidemia and metabolic dysfunction-associated steatotic liver disease (MASLD).

Section	Key Concepts	Pathophysiological Mechanisms	Effect of FIR Therapy	Supporting Evidence
4.1 Gut–Liver Axis and miRNAs	Gut dysbiosis, leaky gut, endotoxemia, miR-122, miR-34a	Activation of TLR4 → hepatic inflammation, insulin resistance; miRNA dysregulation → lipid accumulation and fibrosis	FIR modulates miRNA expression involved in lipid metabolism and inflammation	[91,92]
4.2 Insulin Resistance and Dyslipidemia	Excess FFA, SREBP-1c, ApoB-100, TNF-α, IL-6	↑VLDL production, ↓LDL receptor expression, impaired HDL function → atherogenesis	FIR may enhance insulin sensitivity and reduce lipid abnormalities via AMPK pathway	[93,94,95,96,97,98,99,100,101,102,103,104,105,106,107,108,109]
4.3 Pathogenesis of MASLD	Steatosis → oxidative stress → inflammation → fibrosis → NASH → HCC	Mitochondrial dysfunction, lipid peroxidation, activation of Kupffer and stellate cells	FIR activates AMPK, ↑fatty acid oxidation, ↓lipogenesis gene expression (e.g., CD36, FASN)	[107,108,110,111,112,113,114,115,116]
4.4 Animal Studies	FIR-treated mice (4-week protocol)	↓Hepatic TG and TC, ↓serum LDL-C and TG, ↑HDL-C	Improved lipid profile, reduced hepatic steatosis	[76,117,118]
4.5 Human Clinical Study	4 adults with dyslipidemia (3-week FIR intervention)	↓LDL-C (~13%), ↓TG (~value incomplete), ↑HDL-C	Improved lipid profile	[119,120]

**Table 3:** Recommended safe exposure parameters for infrared light therapies.

Parameter	Recommended Safe Range	Notes
Skin surface temperature	<42 °C	Above 42 °C, risk of burns and tissue damage increases.
Power density (irradiance)	10–100 mW/cm^2^	Higher values increase thermal load; individual tolerance varies.
Session duration	15–30 min per session	Sessions longer than 30 min may increase dehydration and thermal risk.
Treatment frequency	3–5 times per week	Adjusted based on clinical response and tolerance.
Distance from emitter	20–50 cm from the skin surface	Too close proximity increases local heating and burn risk.
Use of protective measures	Protective eyewear, hydration before/after	Eye protection and hydration are strongly recommended.

## Data Availability

No new data were created or analyzed in this study. Data sharing is not applicable to this article.
